# Development of a LAMP-on-Chip Assay for Simultaneous Detection of Mealybugs and Plant Viruses

**DOI:** 10.3390/insects17070731

**Published:** 2026-07-16

**Authors:** Jiaying Wang, Junxia Cui, Li Liu, Xianfeng Chen, Guozhou Cao

**Affiliations:** 1 Technical Center, Ningbo Customs, Ningbo 315100, China; wangjy877@163.com (J.W.); flyingcjx@163.com (J.C.); 2 Technical Center, Ningbo Academy of Inspection and Quarantine, Ningbo 315012, China; 3 Ningbo Zhongsheng Product Testing Co., Ltd., Ningbo 315048, China; liuli545@126.com; 4 Ningbo Customs, Ningbo 315012, China; cxfxhy17@163.com

**Keywords:** microfluidic chip, biosecurity, on-site diagnostics, rapid screening, quarantine pests

## Abstract

Global trade faces increasing biosecurity threats from pests like mealybugs and plant viruses, yet conventional detection methods are usually time-consuming and lab-dependent, creating an urgent need for an efficient portable platform. This study developed a microfluidic chip-based assay to simultaneously identify two mealybug species (*Planococcus minor* and *Dysmicoccus neobrevipes*) and *Orthotospovirus tomatomaculae* (tomato spot wilt virus, TSWV). Species-specific primers targeting conserved genetic regions (*28S rRNA*, *internal transcribed spacer ITS*, and nucleocapsid protein gene) were designed. The assay achieved a limit of detection (LOD) of 10^3^ copies/µL (based on plasmids) and 2.30 × 10^−3^ ng/µL (based on real samples) for *P. minor*, 10^4^ copies/µL and 1.16 × 10^−2^ ng/µL for *D. neobrevipes*, and 10^3^ copies/µL and 2.07 × 10^−2^ ng/µL for TSWV, with no cross-reactivity against non-target species and good producibility both intrassay and interassay. Parallel detection was also successful with complex nucleic acid mixtures. The clinical specificity and sensitivity was 100% and 92.31%. Integrated with isothermal amplification and microfluidic pattern, the quadruple-sector chip enables on-site screening of a maximum of eight samples within 30–60 min. This rapid, sensitive, and portable tool overcomes limitations of conventional phytosanitary methods, supporting real-time customs monitoring and risk-based biosecurity measures.

## Simple Summary

With the continuous expansion of global trade, invasive pest issues have become increasingly prominent. Mealybugs and plant viruses represent significant categories of plant pests, capable of causing ecological imbalances and substantial economic losses. *Planococcus minor*, *Dysmicoccus neobrevipes*, and *Orthotospovirus tomatomaculae* (tomato spot wilt virus, TSWV) are three species in the List of Quarantine Pests for Import Plants of the People’s Republic of China, and thus hold considerable quarantine significance. Traditional detection methods like conventional polymerase chain reaction (PCR) are either inefficient or require laboratory equipment, making them unsuitable for quick inspections at ports. To address this challenge, we developed a microfluidic chip-based screening assay that can rapidly and accurately detect multiple pests and viruses at the same time. This assay is designed to identify two mealybug species (*P. minor* and *D. neobrevipes*) and one plant virus, TSWV. Using species-specific primers and isothermal amplification, this method delivers sensitive, specific, and rapid results within 30–60 min, without the need for complex lab facilities. This technology offers a promising solution for on-site biosecurity monitoring, helping to protect agricultural ecosystems and support efficient, science-based quarantine policies.

## 1. Introduction

The global trade of plants and plant products is increasingly threatened by invasive pests, including mealybugs and plant viruses, which pose serious risks to agricultural ecosystems, economic stability, and international market access [1]. Traditional detection methods, such as morphological identification and conventional molecular diagnostics, are labor-intensive, time-consuming, and laboratory-dependent, making them unsuitable for rapid on-site quarantine inspections [2]. This limitation highlights the urgent need for innovative diagnostic tools capable of efficient and real-time identification of multiple pests and pathogens at ports.

*Planococcus minor* (Hemiptera: Pseudococcidae) represents an important quarantine pest with a broad host range, affecting more than 250 plant species including many economically significant tropical fruits [3]. *Dysmicoccus neobrevipes* (Hemiptera: Pseudococcidae), normally called gray pineapple mealybug, is also a serious quarantine pest infesting tropical fruits like bananas and pineapples [4]. Meanwhile, mealybugs are natural vectors of several plant viruses, such as *Velarivirus arecae* (areca palm velarivirus 1, APV1) [5] and pineapple mealybug wilt-associated viruses (PMWaVs) [6]. *Orthotospovirus tomatomaculae* (Elliovirales: Tospoviridae, tomato spot wilt virus, TSWV) is a damaging quarantine virus with an outstanding host range covering over 1000 plant species [7]. These three organisms are vital targets in port inspection of agricultural products, which attract increasing attention globally.

The DNA barcoding technique is often used for identification of mealybugs, and lacks portability and efficiency [8]. A rapid and sensitive Recombinase Polymerase Amplification (RPA) assay has been applied for *D. neobrevipes* [4]. However, the RPA assay is costly. Real-time reverse transcription loop-mediated isothermal amplification assay developed earlier for TSWV still depends on lab instruments [9]. Furthermore, artificial intelligence (AI) has been utilized in the detection of TSWV infection [10,11]. But the overall accuracy needs to be improved. Moreover, multiplex detection methods based on droplet digital PCR and conventional PCR have been established for mealybugs [12,13]. Yet those multiplex protocols also lack cost effectiveness and portability for complex field applications, especially in resource-limited environments. Recent advances in microfluidic technologies [14] and isothermal amplification techniques offer promising solutions for decentralized and simultaneous detection [15]. These innovations have the potential to significantly enhance diagnostic capabilities in quarantine settings.

This study presents a microfluidic chip-based detection assay designed for simultaneous identification of two high-risk mealybug species and one plant virus. By integrating target-specific primers with automated microfluidic processing, the proposed system aims to overcome the limitations of conventional methods, enabling rapid, high-throughput, and accurate on-site screening without compromising analytical performance.

The urgency of this work is underscored by growing demand for real-time biosecurity measures in global agricultural trade. Climate change has expanded the geographic range of many pests, while increasing import volumes further challenge existing phytosanitary systems [16,17,18,19]. Current protocols are insufficient to address these emerging threats, emphasizing the critical need for innovative tools that align with modern quarantine requirements. This study addresses that need by developing a portable, sensitive, and simultaneous detection platform, thereby strengthening biosecurity resilience and supporting sustainable agricultural trade practices.

## 2. Materials and Methods

### 2.1. Sample Collection

Samples used in this study were obtained from multiple sources, including laboratory reserves (collected at Beilun, Meishan, and Daxie ports, Ningbo, China), institutional donations, and commercial suppliers. A total of 30 *P. minor*, 25 *D. neobrevipes*, and 15 *Ranunculus asiaticus* samples infected with TSWV were collected. Additionally, 49 non-target samples were included for specificity testing, comprising 39 arthropod specimens and 10 plant viruses (Supplementary Table S1). Those arthropod specimens comprise species from Pseudococcidae, Scolytinae, Coleoptera and other insect orders. The selection was designed to include both taxonomically related species and those that may be encountered in similar inspection settings.

### 2.2. Nucleic Acid Preparation

Total DNA was extracted using the UE Genomic DNA Mini Kit (UE Biotech Co., Ltd., Beijing, China) and a quick DNA extraction protocol [20], and total RNA was extracted using the RNeasy Plant Mini Kit (QIAGEN, Hilden, Germany), following the manufacturers’ instructions. Concentrations of all nucleic acid solutions were confirmed via a NanoDrop Spectrophotometer 2000 (Thermo Fisher Scientific, Waltham, MA, USA). Extracted nucleic acids were stored at −20 °C for method development.

For recombinant plasmid construction, genetic regions of three target organisms were synthesized into the pUC57 vector. Plasmids were synthesized by Sangon Biotech (Shanghai) Co., Ltd. (Shanghai, China).

### 2.3. Primer Design and Selection

Multiple alignments of target and non-target species were conducted using MEGA 11 to identify conserved yet species-specific regions [21,22]. These regions were selected for primer design. Specific primers were designed using online tools, including Primer Explorer Version 4 (http://primerexplorer.jp/e, accessed on 18 February 2025) and loop-mediated isothermal amplification (LAMP) Designer (Premier Biosoft, San Francisco, CA, USA) [23]. Primers were synthesized by Sangon Biotech (Shanghai) Co., Ltd. (Shanghai, China).

Primer screening was performed in two steps: firstly with both positive and negative samples, and secondly with positive sample 10-fold dilutions. Primer sets with no false positive/negative amplification, and displaying the lowest time-to-positive (Tp) values, were chosen for further analysis. Sterile ddH_2_O was set as the blank control. Each treatment was conducted in quadruplicate.

### 2.4. Microfluidic Chip-Based Assay

The dished microfluidic chip used in this study consisted of four sectors. Each sector had two separate units, with each unit containing four reaction wells and one loading chamber. These reaction wells were connected with the loading chamber by capillary channels and ball valves. The reaction mixture was loaded via an inlet hole into the chamber [24].

Prior to reaction, 2 μL of primer mix was added to each reaction well and dried at 60 °C for 10 min. Final concentration was 0.2 μM for each of primer F3 and B3, 1.6 μM for each of primer FIP and BIP, and 0.8 μM for LB. Primer concentrations were kept identical for all targets. The chip was then sealed with an adhesive film. Each unit was set for simultaneous detection of three targets in one loading. Primer layout was as follows: one well for *P. minor*, one for *D. neobrevipes*, one for TSWV, and the last one for the blank control. For the other units on the chip, primer layout were the same, making it a maximum of eight different samples analyzed on one chip.

For each loading, 35 μL isothermal amplification master mix (XinVita Bio, Shanghai Xinyi Biotechnology Co., Ltd. (Shanghai, China)) and 5 μL nucleic acid template were mixed and added into the chamber, making a total volume of 40 μL. Reaction mixture was centrifuged into four wells via capillary channels, resulting in 10 μL for each well. The loaded chip was then sealed with aluminum film [25,26].

The chip was placed into the microfluidic detection system (MA2000 Plus, Ningbo iGene Technology Co., Ltd. (Ningbo, China)), and incubated at 63.5 °C for 60 min [24].

### 2.5. Sensitivity of the Microfluidic Chip-Based Assay

The sensitivity of this microfluidic chip-based assay was determined using serial dilutions of recombinant plasmids along with real samples. Sterile ddH_2_O was set as the blank control. Each treatment was conducted in quadruplicate.

### 2.6. Specificity of the Microfluidic Chip-Based Assay

The specificity of microfluidic chip-based assay was analyzed using 14 non-target organisms, including 7 arthropod samples and 7 plant virus samples (Table 1). The concentrations of all tested nucleic acid samples were kept the same (near 2 ng/μL). Sterile ddH_2_O was set as the blank control. Each treatment was conducted in quadruplicate.

### 2.7. Reproducibility of the Microfluidic Chip-Based Assay

The reproducibility of the microfluidic chip-based assay was determined using recombinant plasmids of each target organism at concentrations of 10^6^ copies/μL and 10^4^ copies/μL. Nucleic acid dilutions from real samples (10-fold of limit of detection, LOD) were also used for intra/interassay repeatability analysis. Each treatment was conducted in quadruplicate. Interassay testing was conducted by repeating the experiment three times. In addition, nucleic acid solutions from real samples stored at −20 °C for one year were used to verify the stability of this assay.

### 2.8. Parallel Detection of Multiple Targets

To better describe the ability of this microfluidic chip-based assay in the detection of multiple targets simultaneously, the recombinant plasmid mixed solution was used, including *P. minor* and *D. neobrevipes*, with each concentration at 10^5^ copies/µL. Sterile ddH_2_O was set as the blank control. Each treatment was conducted in quadruplicate.

### 2.9. Conventional Molecular Analysis

Each nucleic acid sample was validated via DNA barcoding or conventional molecular methods. For arthropod samples, the universal *cytochrome c oxidase subunit I* (*COI*) primer set [27] was used for nested PCR. PCR products were sequenced and analyzed using BLAST to confirm identity.

For TSWV, reverse transcription PCR outlined in the Chinese standard GB/T 28982-2012 [28] was utilized. *Nepovirus nicotianae* was confirmed by reverse transcription PCR in the Chinese standard SN/T 1146.2-2017 [29]. *Potyvirus plumpoxi* was diagnosed using methods from the Chinese standard GB/T 31800-2015 [30]. *Nepovirus nigranuli* and *Nepovirus lycopersici* were identified with the Chinese standards GB/T 31795-2015 [31] and SN/T 2670-2010 [32], respectively. *Tobamovirus viridimaculae*, *Comovirus siliquae*, *Tobamovirus fructirugosum*, and *Orthotospovirus impatiensnecromaculae* were diagnosed via these Chinese standards GB/T 28071-2011 [33], GB/T 28063-2011 [34], GB/T 45589-2025 [35], and SN/T 3674-2013 [36], respectively.

Sequencing was performed by Tsingke Biological Technology Co., Ltd. (Beijing, China).

### 2.10. Clinical Sensitivity and Specificity

Conventional molecular diagnostics were set as the standard for clinical sensitivity and specificity analysis of this microfluidic chip-based assay. Crude extractions of real samples were also included as test samples. The clinical sensitivity was calculated as below: clinical sensitivity = true positives/(true positives + false negatives) × 100%. The clinical specificity was inferred as follows: clinical specificity = true negatives/(true negatives + false positives) × 100%. To better describe the consistency between this microfluidic chip-based assay and conventional molecular methods, Kappa value was calculated using SPSS (IBM SPSS Statistics 26).

## 3. Results

### 3.1. Selection of Gene Markers and Primer Sets

**Figure 1:**
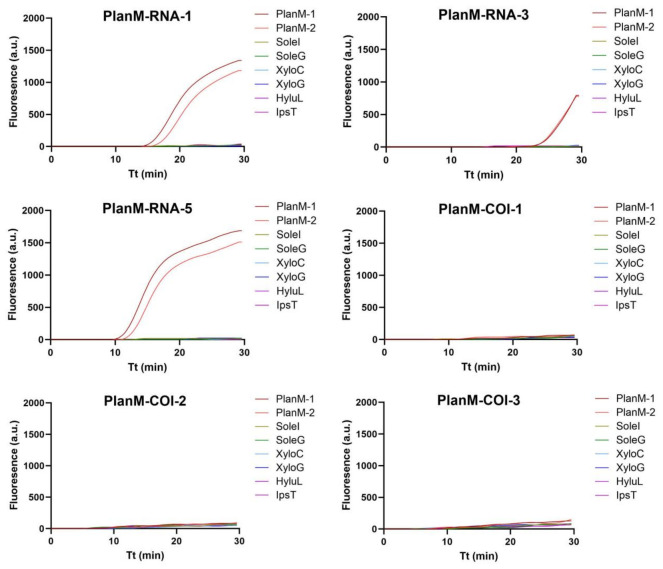
Results of first step of primer selection for *P. minor*. Tt is the time for amplification. Two positive samples (PlanM-1 and PlanM-2) and six negative ones (SoleI, SoleG, XyloC, XyloG, HyluL, and IpsT) were used. Each treatment was conducted in quadruplicate.

**Figure 2:**
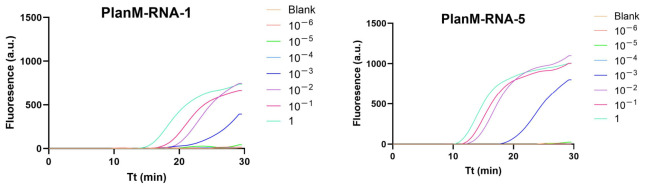
Results of second step of primer selection for *P. minor.* Tt is the time for amplification. Sample PlanM-1 (original concentration: 2.45 ng/µL) was diluted in a 10-fold gradient and used for screening. Sterile ddH_2_O was set as the blank control. Each treatment was conducted in quadruplicate.

Two loci of *P. minor* were selected as alternatives: the *28S rRNA* gene (GenBank accession number: KP692408.1) and the *COI* gene (accession number: KY372784.1). A total of six primer sets were designed and named as follows: PlanM-RNA-1, PlanM-RNA-3, and PlanM-RNA-5 according to the *28S rRNA* gene, and PlanM-COI-1, PlanM-COI-2, and PlanM-COI-3 according to the *COI* gene. The microfluidic chip-based assay was conducted with two positive samples (PlanM-1 and PlanM-2) and six negative ones (SoleI, SoleG, XyloC, XyloG, HyluL, and IpsT). According to the results, PlanM-RNA-1, PlanM-RNA-3, and PlanM-RNA-5 had amplification curves, while the other three primer sets based on the *COI* gene failed with no amplification. Tp values of PlanM-RNA-1, PlanM-RNA-3, and PlanM-RNA-5 were near 15, over 20, and close to 10, respectively (Figure 1). Sample PlanM-1 (original concentration: 2.45 ng/µL) was diluted in a 10-fold gradient and used for further screening. Both PlanM-RNA-1 and PlanM-RNA-5 were able to detect solutions diluted up to 1000-fold (2.45 pg/µL), and PlanM-RNA-5 exhibited lower Tp values (four near 10, and one close to 20). Meanwhile, most Tp values for PlanM-RNA-1 were over 15 (Figure 2). Therefore, PlanM-RNA-5 (Table 2) was selected for further development.

**Figure 3:**
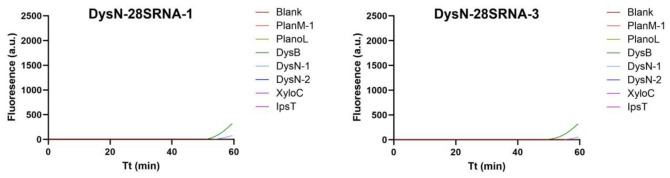

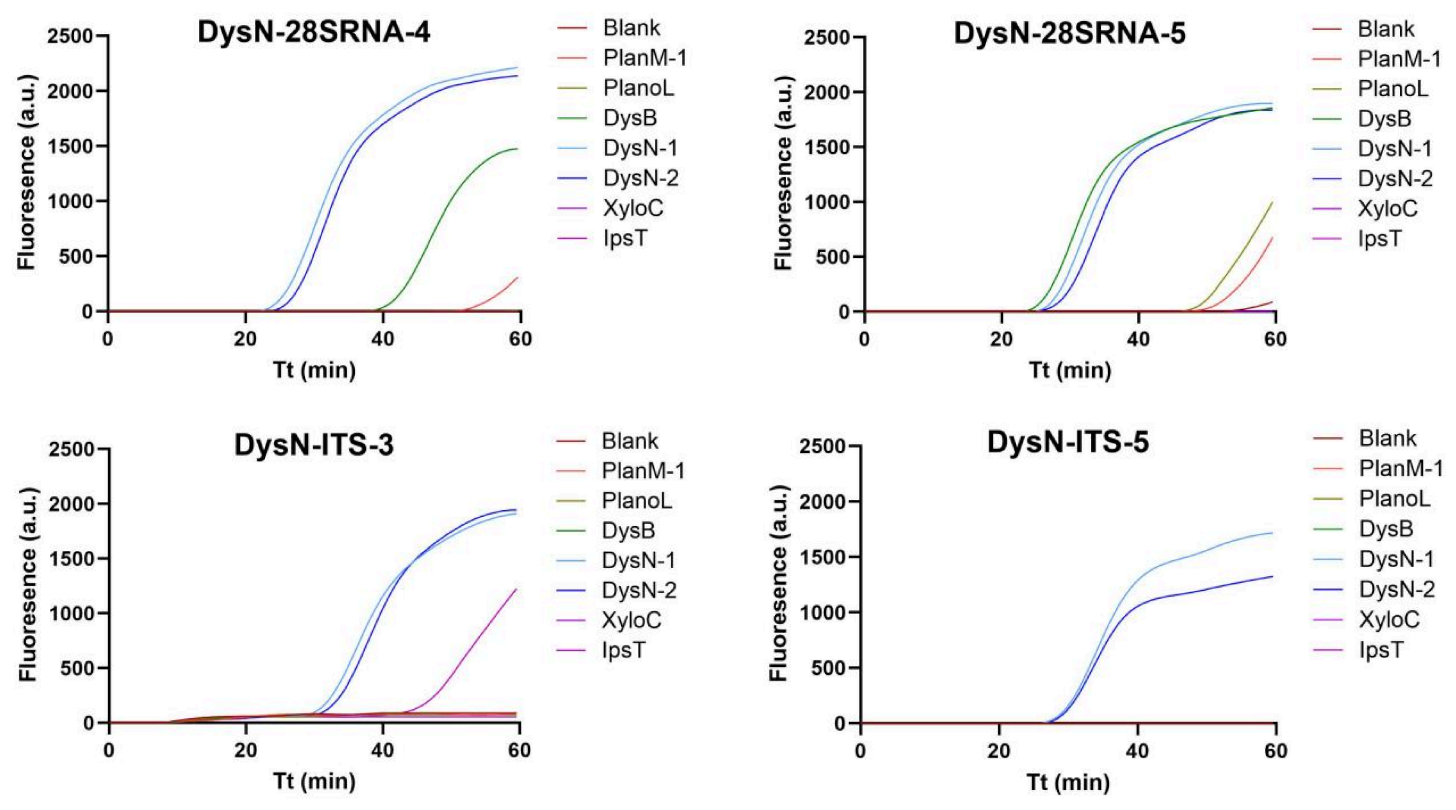
Results of primer selection for *D. neobrevipes*. Tt is the time for amplification. Two positive samples (DysN-1, DysN-2) and 5 negative samples (PlanM-1, PlanoL-1, DysB, XyloC and IpsT) were used. Sterile ddH_2_O was set as the blank control. Each treatment was conducted in quadruplicate.

Two loci of *D. neobrevipes* were selected as targets: the *28S rRNA* gene (accession number: AY427411.1) and the *internal transcribed spacer*, *ITS* gene (accession number: JQ277218.1). Six primer sets were designed and named as follows: DysN-28SRNA-1, DysN-28SRNA-3, DysN-28SRNA-4, and DysN-28SRNA-5 according to the *28S rRNA* gene, and DysN-ITS-3 and DysN-ITS-5 according to the *ITS* gene. Seven samples were used for primer screening: two positive (DysN-1, DysN-2) and five negative (PlanM-1, PlanoL, DysB, XyloC and IpsT). Either DysN-28SRNA-1 or DysN-28SRNA-3 failed in amplification, and DysN-28SRNA-4 showed false amplification for *D. brevipes*. DysN-28SRNA-5 had false amplification for *D. brevipes*, *Ips typographus*, and *P. lilacinus*, and DysN-ITS-3 for *I. typographus*. DysN-ITS-5 displayed a sigmoidal amplification only for two positive samples with a Tp value over 20 (Figure 3). Then DysN-ITS-5 (Table 2) was selected for microfluidic chip-based assay development.

**Figure 4:**
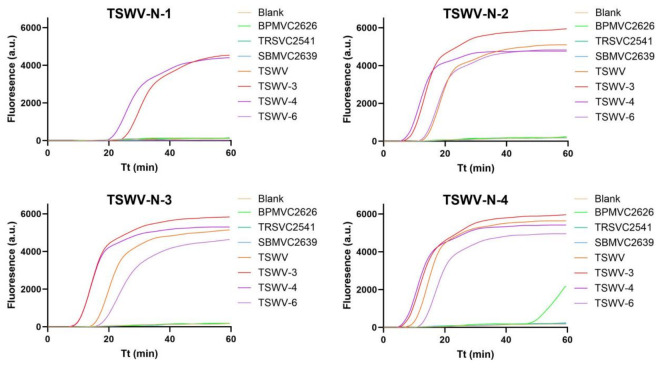
Results of first step of primer selection for TSWV. Tt is the time for amplification. Seven samples (BPMV, TRSV, SBMV, TSWV, TSWV-3, TSWV-4, and TSWV-6) were used. Sterile ddH_2_O was set as the blank control. Each treatment was conducted in quadruplicate.

**Figure 5:**
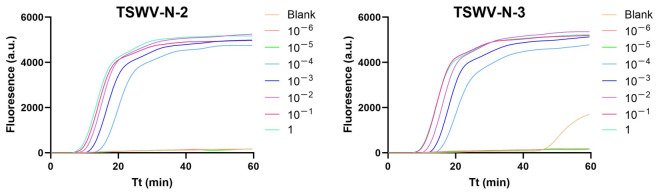
Results of second step of primer selection for TSWV. Tt is the time for amplification. Sample TSWV-3 (original concentration: 207.2 ng/µL) was diluted in a 10-fold gradient and used for screening. Sterile ddH_2_O was set as the blank control. Each treatment was conducted in quadruplicate.

As for TSWV, four primer sets, TSWV-1, TSWV-2, TSWV-3, and TSWV-4, were designed based on the nucleocapsid protein gene (accession number: HQ406981.1) [37]. These primers were screened using seven samples (BPMV, TRSV, SBMV, TSWV, TSWV-3, TSWV-4, and TSWV-6). Based on the results, TSWV-N-1 only amplified two positive samples (TSWV and TSWV-4), with the other two being false negatives. TSWV-N-2 and TSWV-N-3 amplified all four positive samples, while no amplification was observed with three negative ones or distilled water. TSWV-N-4 gave a false amplification for BPMV (Figure 4). Therefore, the primers TSWV-N-2 and TSWV-N-3 were selected for subsequent analysis. The sample TSWV-3 (original concentration: 207.2 ng/µL) was diluted in a 10-fold gradient and used for further screening. Results showed that the primer TSWV-N-2 was able to detect sample solution diluted up to 10,000-fold (20.72 pg/µL), while TSWV-N-3 gave a weak false amplification for the blank control (Figure 5). Thus, the primer TSWV-N-2 (Table 2) was selected for the development of a microfluidic chip-based assay.

### 3.2. Analysis of the Sensitivity of Microfluidic Chip-Based Assay

**Figure 6:**
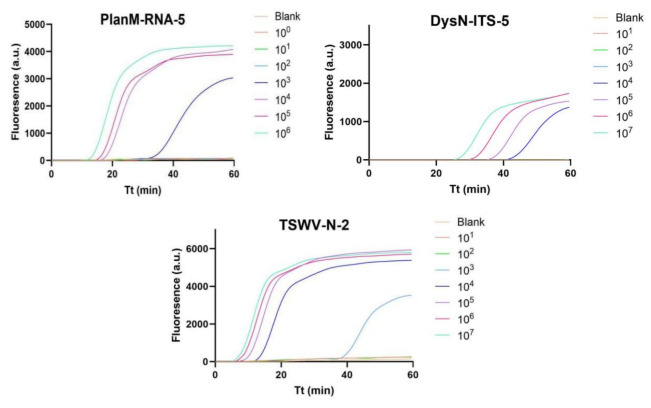
Results of sensitivity analysis based on recombinant plasmids. Tt is the time for amplification. Solutions 10^1^–10^7^ correspond to concentrations of 10^1^–10^7^ copies/μL. Sterile ddH_2_O was set as the blank control. Each treatment was conducted in quadruplicate.

The sensitivity of this microfluidic chip-based assay was evaluated using synthesized plasmids containing target regions from *P. minor*, *D. neobrevipes*, and TSWV, respectively. Plasmid dilutions were prepared in a 10-fold gradient. Corresponding results showed that the PlanM-RNA-5 reaction system produced ‘S’ curves for the plasmid of *P. minor* at the concentrations of 10^6^ copies/µL, 10^5^ copies/µL, 10^4^ copies/µL, and 10^3^ copies/µL, while no amplification was achieved at 10^2^ copies/µL or below. As with DysN-ITS-5, concentrations of *D. neobrevipes* plasmid at 10^7^ copies/µL, 10^6^ copies/µL, 10^5^ copies/µL, and 10^4^ copies/µL had positive results, yet other concentrations had negative outcomes. For TSWV-N-2, TSWV plasmid concentrations at 10^7^ copies/µL, 10^6^ copies/µL, 10^5^ copies/µL, 10^4^ copies/µL, and 10^3^ copies/µL displayed typical amplification curves, with others being flat (Figure 6).

**Figure 7:**
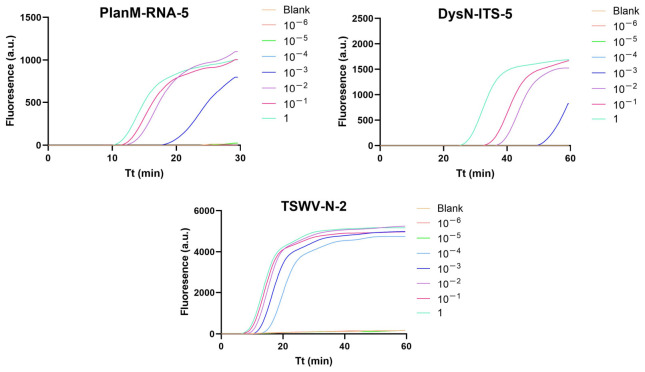
Results of sensitivity analysis based on real samples. Tt is the time for amplification. PlanM-2 (original concentration: 2.30 ng/µL), DysN-1 (original concentration: 11.60 ng/µL), and TSWV-3 (original concentration: 207.2 ng/µL) were used for PlanM-RNA-5, DysN-ITS-5, and TSWV-N-2, respectively. Solution 10^−1^ means 10-fold dilution of the original. Solution 10^−2^ means 100-fold dilution of the original. The rest dilutions were made in the same manner. Sterile ddH_2_O was set as the blank control. Each treatment was conducted in quadruplicate.

Nucleic acid solutions extracted from real samples were also utilized in sensitivity analysis, diluted in 10-fold. PlanM-2 (original concentration: 2.30 ng/µL), DysN-1 (original concentration: 11.60 ng/µL), and TSWV-3 (original concentration: 207.2 ng/µL) were used for LOD evaluation for reaction systems of PlanM-RNA-5, DysN-ITS-5, and TSWV-N-2, respectively. For PlanM-RNA-5, PlanM-2 concentrations at 2.30 ng/µL, 2.30 × 10^−1^ ng/µL, 2.30 × 10^−2^ ng/µL, and 2.30 × 10^−3^ ng/µL displayed amplification curves, while concentrations at 2.30 × 10^−4^ ng/µL or below were negative. For DysN-ITS-5, DysN-1 dilutions of 11.60 ng/µL, 1.16 ng/µL, 1.16 × 10^−1^ ng/µL, and 1.16 × 10^−2^ ng/µL were positive, with others being flat. For TSWV-N-2, TSWV-3 solutions at the concentration of 207.2 ng/µL, 20.72 ng/µL, 2.07 ng/µL, 2.07 × 10^−1^ ng/µL, and 2.07 × 10^−2^ ng/µL achieved amplification, yet other solutions failed (Figure 7).

### 3.3. Analysis of the Specificity of Microfluidic Chip-Based Assay

**Figure 8:**
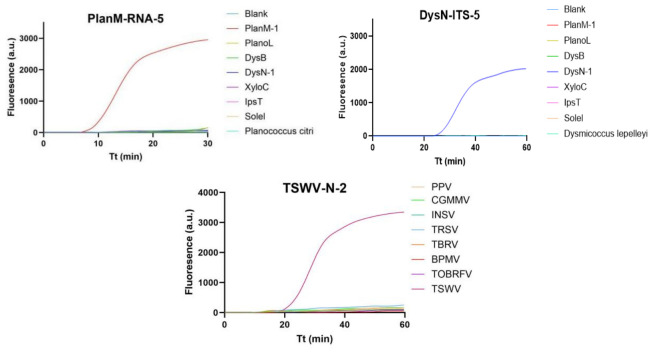
Results of specificity analysis. Tt is the time for amplification. Seventeen samples were used, including PlanM-1, PlanoL, DysB, DysN-1, XyloC, IpsT, SoleI, and Planococcus citri for PlanM-RNA-5; PlanM-1, PlanoL, DysB, DysN-1, XyloC, IpsT, SoleI, and Dysmicoccus lepelleyi for DysN-ITS-5; and PPV, CGMMV, INSV, TRSV, TBRV, BPMV, ToBRFV, and TSWV for TSWV-N-2. Sterile ddH_2_O was set as the blank control. Each treatment was conducted in quadruplicate.

Seventeen samples were used to evaluate the specificity of this assay, including: PlanM-1, PlanoL, DysB, DysN-1, XyloC, IpsT, SoleI, Planococcus citri, and Dysmicoccus lepelleyi (for reaction systems of PlanM-RNA-5 and DysN-ITS-5), and PPV, CGMMV, INSV, TRSV, TBRV, BPMV, ToBRFV, and TSWV (for reaction system of TSWV-N-2). According to the results, only PlanM-1, DysN-1, and TSWV showed typical positive amplification with reaction systems of PlanM-RNA-5, DysN-ITS-5, and TSWV-N-2, respectively. Thus, no cross-reactivity was observed with non-target species, indicating the strong specificity of this microfluidic chip-based assay (Figure 8).

### 3.4. Analysis of the Reproducibility of Microfluidic Chip-Based Assay

**Figure 9:**
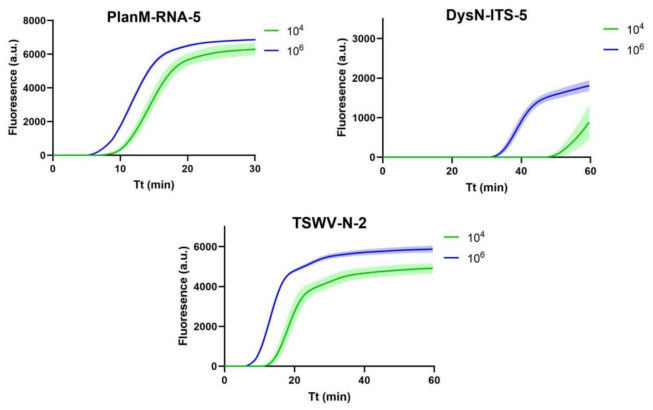
Results of repeatability test based on synthesized plasmids. Tt is the time for amplification. Synthesized plasmids (concentration: 10^6^ copies/μL and 10^4^ copies/μL) were used. Color shadow corresponds to CV value. A wider shadow means a bigger CV value. Each treatment was conducted in quadruplicate.

**Figure 10:**
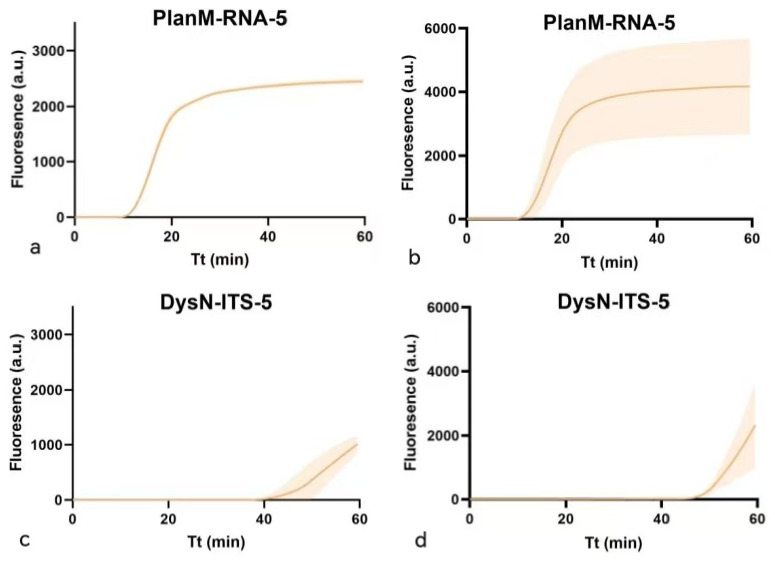

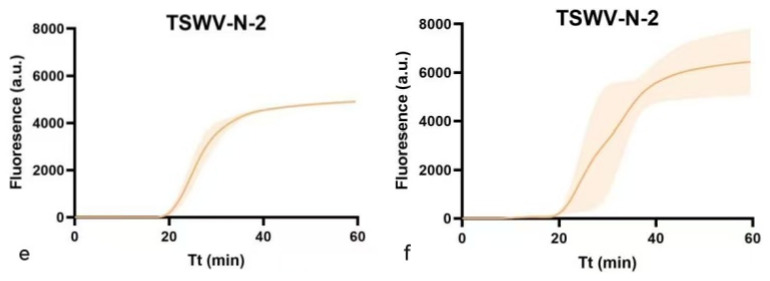
Results of repeatability test based on real samples. Tt is the time for amplification. Color shadow corresponds to CV value. A wider shadow means a bigger CV value. (**a**,**c**,**e**): Intra-assay; (**b**,**d**,**f**): interassay. Nucleic acid dilutions from real samples (10-fold of limit of detection, LOD) were used for intra-/interassay repeatability analysis. Each treatment was conducted in quadruplicate. Interassay test was conducted by repeating the experiment three times.

In this study, synthesized plasmids (concentration: 10^6^ copies/μL and 10^4^ copies/μL) and real sample solutions (10-fold of LOD) of three target organisms were used for reproducibility testing. Results showed that the coefficient of variation (CV) for PlanM-RNA-5 was 5.567% for plasmid dilutions at 10^4^ copies/μL and 4.591% at 10^6^ copies/μL. The CV for DysN-ITS-5 was 2.210% at 10^4^ copies/µL and 1.615% at 10^6^ copies/µL. For TSWV-N-2, the CV was 6.933% at 10^4^ copies/µL and 3.154% at 10^6^ copies/µL (Figure 9). As with real samples, the CV for PlanM-RNA-5 was 2.427% intra-assay and 8.422% interassay both at the concentration of 2.45 × 10^−2^ ng/µL. For DysN-ITS-5, the CV was 7.549% intra-assay and 1.448% interassay at 1.16 × 10^−1^ ng/µL. For TSWV-N-2, the CV was 3.829% intra-assay and 16.324% interassay at 2.07 × 10^−1^ ng/µL (Figure 10).

**Figure 11:**
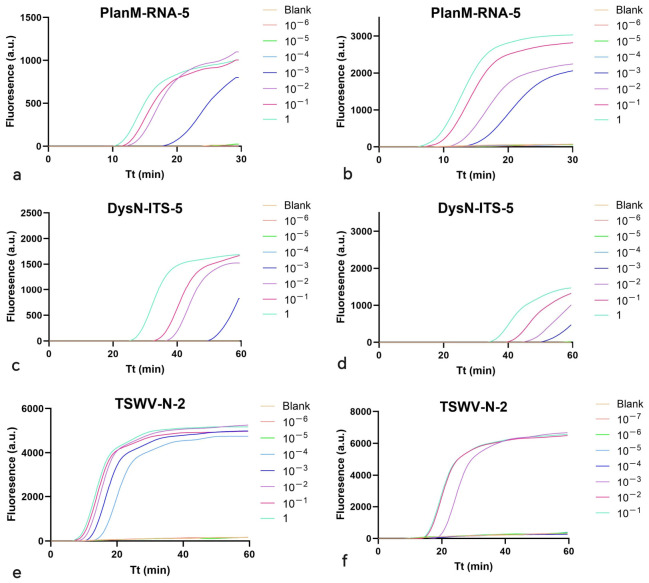
Results of stability analysis based on real samples. Tt is the time for amplification. (**a**,**c**,**e**): Results right after extraction; (**b**,**d**,**f**): results after one year storage at −20 °C. PlanM-2 (original concentration: 2.30 ng/µL), DysN-1 (original concentration: 11.60 ng/µL), and TSWV-3 (original concentration: 207.2 ng/µL) were used for PlanM-RNA-5, DysN-ITS-5, and TSWV-N-2, respectively. Further, 10^−1^ means 10-fold dilution of the original solution; 10^−2^ means 100-fold dilution of the original solution. The remaining dilutions were made in the same manner. Sterile ddH_2_O was set as the blank control. Each treatment was conducted in quadruplicate.

Nucleic acid solutions from real samples stored at −20 °C for one year were utilized for the stability test. For PlanM-RNA-5, Tp values displayed little difference after one year when diluted to 1000-fold, with all below 20 for four positive concentrations. For DysN-ITS-5, Tp became higher after one year when diluted to 1000-fold. For TSWV-N-2, dilution at 10000-fold was flat while dilutions at 1000-fold and above showed positive amplification curves (Figure 11). Thus, RNA was more likely to be degraded than DNA.

### 3.5. Parallel Detection of Multiple Targets

**Figure 12:**
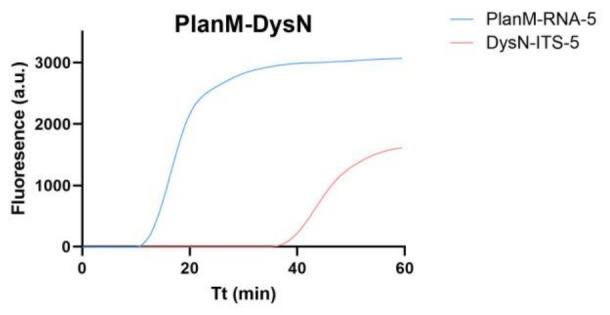
Results of parallel detection of plasmid mixture. Tt is the time for amplification. Recombinant plasmid mixed solution was used, including *P. minor* and *D. neobrevipes*, with each concentration at 10^5^ copies/µL. Sterile ddH_2_O was set as the blank control. Each treatment was conducted in quadruplicate.

The plasmid combination of *P. minor* and *D. neobrevipes* displayed expected amplification, with the Tp of the former below 20 and the other near 40 (Figure 12). Though complex nucleic acid solutions may affect the Tp value, positive feedback could still be achieved.

### 3.6. Clinical Sensitivity and Specificity of the Microfluidic Chip-Based Assay

Among samples collected previously, 29 showed positive results for *P. minor*, 21 positive for *D. neobrevipes*, and 10 positive for TSWV (Supplementary Table S1) via conventional molecular methods. Of these 29 nucleic acid samples of *P. minor*, 5 were crude extractions. For *D. neobrevipes,* 6 were crude extractions among 21 samples. Microfluidic chip-based assay detected 29 out of 29 for *P. minor*, 21 of 21 for *D. neobrevipes*, and 10 of 10 for TSWV. Then the clinical sensitivity was 100% for each of three reaction systems. For those negative samples, PlanM-RNA-5 and TSWV-N-2 showed expected results, resulting in a clinical specificity of 100%. As with DysN-ITS-5, negative results were observed for only 36 out of 39 samples, which resulted in a clinical specificity of 92.31%. The Kappa values maintained were 1.000, 0.632, and 1.000 for reaction systems of PlanM-RNA-5, DysN-ITS-5, and TSWV-N-2, respectively (Table 3). These data suggest good specificity and sensitivity of the microfluidic chip-based assay, and crude extractions of bugs could also be screened successfully.

## 4. Discussion

Rapid detection protocols based on nucleic acid isothermal amplification have been increasingly applied in plant inspection at ports [38,39,40]. Portable datum-analysis devices boost the paradigm shift in phytosanitary measures, representing rapid, point-of-care screening of pests with accuracy and minimal expertise [41,42,43]. This study presents a microfluidic chip-based LAMP assay for simultaneous screening of three targets including two mealybugs and one plant virus, which are often encountered at ports during inspection of agricultural crops. Combined with a rapid nucleic acid extraction protocol, it can hopefully shorten the screening time to less than 1.5 h. Crude extractions from mealybugs were tested in this study, which gave the expected results. In addition, this chip-based assay allows eight different samples to be screened on a single chip at the same time. With the maturation of microfluidic chip technology and commercial promotion of related instruments along with reagents, the cost of one reaction has been reduced to nearly $ 2.96. The cost of the microfluidic detection system (MA2000 Plus) is nearly $ 30,000. In this study, the device was used for free, because of the long-term purchase orders for reagents and consumables with the suppliers. Thus, this study demonstrates the technical and economic feasibility of this assay for routine phytosanitary applications at ports, which can enhance breadth and efficiency of screening, offering value by addressing the challenge of diverse and potentially co-occurring invasive plant pests and diseases.

The *28S rRNA* gene, *COI* gene, and *ITS* gene are commonly used molecular markers for mealybug identification [44]. In this study, these three markers were all considered potential genetic targets and corresponding primers were designed. PlanM-COI-1, PlanM-COI-2, and PlanM-COI-3 failed in the amplification of positive samples for *P. minor*. DysN-28SRNA-1 and DysN-28SRNA-3 displayed false negative results, while DysN-28SRNA-4 and DysN-28SRNA-5 showed false positives for non-target organisms. Only one loop primer (LB) was designed for each of these three targets due to sequence constraints.

LOD is an important criteria for evaluating a detection method. In this study, the LOD of microfluidic chip-based assay was assessed using both plasmids and real samples. For PlanM-RNA-5, the LODs were calculated to be 10^3^ copies/µL based on plasmids and 2.30 × 10^−3^ ng/µL based on real samples. For DysN-ITS-5, the LODs were 10^4^ copies/µL based on plasmids and 1.16 × 10^−2^ ng/µL based on real samples. For TSWV-N-2, values were 10^3^ copies/µL and 2.07 × 10^−2^ ng/µL. A LOD of 4 × 10^−5^ ng/µL was reported earlier for the LAMP method for *Alternaria* spp., which was nearly 100 times more sensitive than PCR [34]. In our study, the LODs of the microfluidic chip-based assay were similar to those of PCR.

For reproducibility analysis, both synthesized plasmids (concentration: 10^6^ copies/μL and 10^4^ copies/μL) and real sample solutions (10-fold of LOD) of three target organisms were used. CV values were all below 10% except the interassay one for the TSWV-N-2 reaction system. The reason could be related to the heterogeneity and instability of RNA solutions. In addition, nucleic acid solutions extracted from real samples could still be detected after one year of storage at −20 °C, except for the RNA solutions aimed at TSWV, which were diluted to 10,000-fold and below. It is reasonable that RNA is more easily degraded than DNA. Thus, RNA solutions for virus screening should be conducted as soon as possible.

Parallel detection was also confirmed with mixed plasmid solutions, which demonstrates the efficiency of this microfluidic chip-based assay from another perspective. The clinical sensitivity and specificity were calculated for these three reaction systems, which turned out to be high except for the clinical specificity of DysN-ITS-5, at 92.31%. The Kappa value with conventional molecular methods fell to 0.632 for DysN-ITS-5, with the other two systems at 1.000. The reason can be traced back to three *Dysmicoccus* sp. samples. Because of the long preservation time, nucleic acid could be degraded and accurate identification could not be settled through DNA barcoding. Thus, the divergence occurred between this assay and conventional molecular methods.

Widespread adoption of this technology still faces several challenges [32]. Currently, the mass production cost of the chips, the need for dynamic updates to target databases in response to evolving pests and disease outbreaks, and mutual recognition of detection results across different platforms are all pressing issues that require solutions. Future research should focus on developing low-cost manufacturing processes, establishing an open mechanism for updating plant quarantine databases, and advancing data fusion algorithms that integrate this technology more deeply with existing customs information systems. The ultimate goal is to transition from a technical tool to a routine regulatory capability.

## Figures and Tables

**Table 1:** Viruses used in specificity analysis.

Abbreviation	Species Name	English Name	Sample	Family, Genus
INSV	*Orthotospovirus impatiensnecromaculae*	impatiens necrotic spot virus	unknown leaf powder (Agdia Inc., Elkhart, IN, USA)	Family: Tospoviridae Genus: *Orthotospovirus*
TRSV	*Nepovirus nicotianae*	tobacco ringspot virus	unknown leaf powder (Agdia Inc., Elkhart, IN, USA)	Family: Secoviridae Genus: *Nepovirus*
PPV	*Potyvirus plumpoxi*	plum pox virus	*Prunus* sp.	Family: Potyviridae Genus: *Potyvirus*
TBRV	*Nepovirus nigranuli*	tomato black ring virus	unknown leaf powder (Agdia Inc., Elkhart, IN, USA)	Family: Secoviridae Genus: *Nepovirus*
ToRSV	*Nepovirus lycopersici*	tomato ringspot virus	unknown leaf powder (Agdia Inc., Elkhart, IN, USA)	Family: Secoviridae Genus: *Nepovirus*
CGMMV	*Tobamovirus viridimaculae*	cucumber green mottle mosaic virus	unknown leaf powder (Agdia Inc., Elkhart, IN, USA)	Family: Virgaviridae Genus: *Tobamovirus*
BPMV	*Comovirus siliquae*	bean pod mottle virus	*Glycine max*	Family: Secoviridae Genus: *Comovirus*
ToBRFV	*Tobamovirus fructirugosum*	tomato brown rugose fruit virus	*Capsicum annuum*	Family: Virgaviridae Genus: *Tobamovirus*

**Table 2:** Primer list.

Species	Primer Name	Sequence (5′-3′)
*Planococcus minor*	PlanM-RNA-5-F3	GTTCGACGGTGCGTGTC
	PlanM-RNA-5-B3	TCCTTGGTCCGTGTTTCAAG
	PlanM-RNA-5-FIP	TCCGGCGAGTCGGCCAGAAGAGTCGTTTCGGCGGCTA
	PlanM-RNA-5-BIP	CGACCTAGCGTCGTCGTTGGACGGGTCGGAAAGAGGC
	PlanM-RNA-5-LB	CCGGTCTGCGACGAATCT
*Dysmicoccus neobrevipes*	DysN-ITS-5-F3	GAACACCGTGTTCGACGAA
	DysN-ITS-5-B3	CACGCGCGAACTGTGTAG
	DysN-ITS-5-FIP	GTATCTACTGCTGCGCGCGCCGTTGCCGCGTAAATGAAC
	DysN-ITS-5-BIP	AATTCTCCACTCGGACGCGCTACCCCGTGTGTCCATCT
	DysN-ITS-5-LB	CGCTACAGAGAATAGACGACGC
*Orthotospovirus tomatomaculae*	TSWV-N-2-F3	ATAGCTTGATTAGGGTCAGG
	TSWV-N-2-B3	AAAGCTATCAACTGAAGCAATA
	TSWV-N-2-FIP	GGTGGGAAGCAATCTTAGATTTGACTTGTTGAGGAAACTGGGA
	TSWV-N-2-BIP	TGATTCAAGCCTATGGATTACCTCTGAGGTAAACTACCTCCTAGCA
	TSWV-N-2-LB	CAAAGTCTGTGAGGCTTGCCAT

**Table 3:** Clinical sensitivity, specificity, and Kappa values of three reaction systems.

	PlanM-RNA-5	DysN-ITS-5	TSWV-N-2
Clinical sensitivity	100% (29/29)	100% (21/21)	100% (10/10)
Clinical specificity	100% (37/37)	92.31% (36/39)	100% (10/10)
Kappa	1.000	0.632	1.000

## Data Availability

The original contributions presented in this study are included in the article/Supplementary Materials. Further inquiries can be directed to the corresponding author.

## Supplementary Materials

The following supporting information can be downloaded at https://www.mdpi.com/article/10.3390/insects17070731/s1, Table S1: Sample list.

## Abbreviations

The following abbreviations are used in this manuscript:

TSWV	Tobacco spot wilt virus
INSV	Impatiens necrotic spot virus
BPMV	Bean pod mottle virus
TRSV	Tobacco ring spot virus
TBRV	Tobacco black ring virus
PPV	Plum pox virus
CGMMV	Cucumber green mottle mosaic virus
ToBRFV	Tomato brown rugose fruit virus
LAMP	Loop-mediated isothermal amplification
F3/B3	Forward outer primer/backward outer primer
FIP/BIP	Forward inner primer/backward inner primer
LB	Loop backward primer
CV	Coefficient of variation
LOD	Limit of detection
ITS	Internal transcribed spacer
