# Supplementary material for: Development of a LAMP-on-Chip Assay for Simultaneous Detection of Mealybugs and Plant Viruses
Source: Insects. 2026 Jul 16;17(7):731. doi: 10.3390/insects17070731 (PMC13409837; doi:10.3390/insects17070731)
Supplement: Supplementary file 1 [file insects-17-00731-s001.zip › insects-4297490-supplementary.pdf]

Table S1. Sample list

No.	Marker	Name	Source	Condition
1	PlanM-1	*Planococcus minor*	sample reserve	Preserved in pure ethanol
2	PlanM-2	*P. minor*	sample reserve	Preserved in pure ethanol
3	PlanM-3	*P. minor*	sample reserve	Preserved in pure ethanol
4	PlanM-4	*P. minor*	sample reserve	Preserved in pure ethanol
5	PlanM-5	*P. minor*	sample reserve	Preserved in pure ethanol
6	PlanM-6	*P. minor*	sample reserve	Preserved in pure ethanol
7	PlanM-7	*P. minor*	sample reserve	Preserved in pure ethanol
8	PlanM-8	*P. minor*	sample reserve	Preserved in pure ethanol
9	PlanM-9	*P. minor*	sample reserve	Preserved in pure ethanol
10	PlanM-C5	*P. minor*	sample reserve	Preserved in pure ethanol
11	PlanM-10	*P. minor*	donated by other labs	Preserved in pure ethanol
12	PlanM-11	*P. minor*	donated by other labs	Preserved in pure ethanol
13	PlanM-12	*P. minor*	donated by other labs	Preserved in pure ethanol
14	PlanM-13	*P. minor*	donated by other labs	Preserved in pure ethanol
15	PlanM-14	*P. minor*	donated by other labs	Preserved in pure ethanol
16	PlanM-15	*P. minor*	donated by other labs	Preserved in pure ethanol
17	PlanM-16	*P. minor*	donated by other labs	Preserved in pure ethanol
18	PlanM-17	*P. minor*	donated by other labs	Preserved in pure ethanol
19	PlanM-18	*P. minor*	donated by other labs	Preserved in pure ethanol
20	PlanM-19	*P. minor*	donated by other labs	Preserved in pure ethanol
21	PlanM-20	*P. minor*	donated by other labs	Preserved in pure ethanol
22	PlanM-21	*P. minor*	donated by other labs	Preserved in pure ethanol
23	PlanM-22	*P. minor*	donated by other labs	Preserved in pure ethanol
24	PlanM-23	*P. minor*	donated by other labs	Preserved in pure ethanol
25	PlanM-24	*P. minor*	donated by other labs	Preserved in pure ethanol
26	PlanM-25	*P. minor*	donated by other labs	Preserved in pure ethanol
27	PlanM-26	*P. minor*	donated by other labs	Preserved in pure ethanol
28	PlanM-27	*P. minor*	donated by other labs	Preserved in pure ethanol
29	PlanM-28	*P. minor*	donated by other labs	Preserved in pure ethanol
30	PlanM-29	*P. minor*	donated by other labs	Preserved in pure ethanol
31	DysN-1	*Dysmicoccus neobrevipes*	sample reserve	Preserved in pure ethanol
32	DysN	*D. neobrevipes*	sample reserve	Preserved in pure ethanol
33	DysN-2	*D. neobrevipes*	sample reserve	Preserved in pure ethanol
34	DysN-4	*D. neobrevipes*	sample reserve	Preserved in pure ethanol
35	DysN-C1	*D. neobrevipes*	sample reserve	Preserved in pure ethanol
36	DysN-C3	*D. neobrevipes*	sample reserve	Preserved in pure ethanol
37	DysN-C4	*D. neobrevipes*	sample reserve	Preserved in pure ethanol
38	DysN-5	*D. neobrevipes*	sample reserve	Preserved in pure ethanol
39	DysN-6	*D. neobrevipes*	sample reserve	Preserved in pure ethanol
40	DysN-7	*D. neobrevipes*	sample reserve	Preserved in pure ethanol
41	DysN-8	*D. neobrevipes*	donated by other labs	Preserved in pure ethanol
42	DysN-9	*D. neobrevipes*	donated by other labs	Preserved in pure ethanol
43	DysN-10	*D. neobrevipes*	donated by other labs	Preserved in pure ethanol
44	DysN-11	*D. neobrevipes*	donated by other labs	Preserved in pure ethanol
45	DysN-12	*D. neobrevipes*	donated by other labs	Preserved in pure ethanol
46	DysN-13	*D. neobrevipes*	donated by other labs	Preserved in pure ethanol
47	DysN-14	*D. neobrevipes*	donated by other labs	Preserved in pure ethanol

No.	Marker	Name	Source	Condition
48	DysN-15	*D. neobrevipes*	donated by other labs	Preserved in pure ethanol
49	DysN-16	*D. neobrevipes*	donated by other labs	Preserved in pure ethanol
50	DysN-17	*D. neobrevipes*	donated by other labs	Preserved in pure ethanol
51	DysN-18	*D. neobrevipes*	donated by other labs	Preserved in pure ethanol
52	DysN-19	*D. neobrevipes*	donated by other labs	Preserved in pure ethanol
53	DysN-20	*D. neobrevipes*	donated by other labs	Preserved in pure ethanol
54	DysN-21	*D. neobrevipes*	donated by other labs	Preserved in pure ethanol
55	DysN-22	*D. neobrevipes*	donated by other labs	Preserved in pure ethanol
56	TSWV-1	*Orthotospovirus tomatomaculae* (*Ranunculus asiaticus*)	sample reserve	store in -80 °C
57	TSWV	*O. Tomatomaculae* (*Ranunculus asiaticus*)	sample reserve	store in -80 °C
58	TSWV-3	*O. Tomatomaculae* (*Ranunculus asiaticus*)	sample reserve	store in -80 °C
59	TSWV-4	*O. Tomatomaculae* (*Ranunculus asiaticus*)	sample reserve	store in -80 °C
60	TSWV-5	*O. Tomatomaculae* (*Ranunculus asiaticus*)	sample reserve	store in -80 °C
61	TSWV-6	*O. Tomatomaculae* (*Ranunculus asiaticus*)	sample reserve	store in -80 °C
62	TSWV-7	*O. Tomatomaculae* (*Ranunculus asiaticus*)	sample reserve	store in -80 °C
63	TSWV-8	*O. Tomatomaculae* (*Ranunculus asiaticus*)	sample reserve	store in -80 °C
64	TSWV-9	*O. Tomatomaculae* (*Ranunculus asiaticus*)	sample reserve	store in -80 °C
65	TSWV-10	*O. Tomatomaculae* (*Ranunculus asiaticus*)	sample reserve	store in -80 °C
66	TSWV-11	*O. Tomatomaculae* (*Ranunculus asiaticus*)	sample reserve	store in -80 °C
67	TSWV-12	*O. Tomatomaculae* (*Ranunculus asiaticus*)	sample reserve	store in -80 °C
68	TSWV-13	*O. Tomatomaculae* (*Ranunculus asiaticus*)	sample reserve	store in -80 °C
69	TSWV-14	*O. Tomatomaculae* (*Ranunculus asiaticus*)	sample reserve	store in -80 °C
70	TSWV-15	*O. Tomatomaculae* (*Ranunculus asiaticus*)	sample reserve	store in -80 °C
71	PlanC	*Planococcus citri*	donated by other labs	Fresh sample
72	DysL	*Dysmicoccus lepelleyi*	donated by other labs	Preserved in pure ethanol
73	SoleI	*Solenopsis invicta*	sample reserve	Preserved in pure ethanol
74	SoleG	*S. geminata*	sample reserve	Preserved in pure ethanol
75	XyloC	*Xylosandrus crassiusculus*	sample reserve	Preserved in pure ethanol
76	XyloG	*X. germanus*	sample reserve	Preserved in pure ethanol
77	HyluL	*Hylurgus ligniperda*	sample reserve	Preserved in pure ethanol
78	IpsT	*Ips typographus*	sample reserve	Preserved in pure ethanol
79	PlanX	*Planococcus sp.*	sample reserve	Preserved in pure ethanol
80	PseuX	*Pseudococcus sp.*	sample reserve	Preserved in pure ethanol
81	XyleS	*Xyleborinus saxesenii*	sample reserve	Preserved in pure ethanol
82	IpsG	*I. grandicollis*	sample reserve	Preserved in pure ethanol

No.	Marker	Name	Source	Condition
83	XyloC-2	*Xylosandrus compactus*	sample reserve	Preserved in pure ethanol
84	BacD	*Bactrocera dorsalis*	sample reserve	Preserved in pure ethanol
85	CoptX	*Coptotermes sp.*	sample reserve	Preserved in pure ethanol
86	DacT	*Dacus trimacula*	sample reserve	Preserved in pure ethanol
87	IpsG	*I. grandicollis*	sample reserve	Preserved in pure ethanol
88	IpsD	*I. dentatus*	sample reserve	Preserved in pure ethanol
89	IpsA	*I. acuminatus*	sample reserve	Preserved in pure ethanol
90	IpsS	*I. sexdentatus*	sample reserve	Preserved in pure ethanol
91	MessB	*Messor barbarus*	sample reserve	Preserved in pure ethanol
92	PogB	*Pogonomyrmex barbatus*	sample reserve	Preserved in pure ethanol
93	WasA	*Wasmannia auropunctata*	sample reserve	Preserved in pure ethanol
94	AttM	*Atta mexicana*	sample reserve	Preserved in pure ethanol
95	HolX	*Holotrichia sp.*	sample reserve	Preserved in pure ethanol
96	ErtF	*Erthesina fullo*	sample reserve	Preserved in pure ethanol
97	AchD	*Acheta domesticus*	sample reserve	Preserved in pure ethanol
98	ProT	*Prostephanus truncatus*	sample reserve	Preserved in pure ethanol
99	TheO	*Theretra oldenlandiae*	sample reserve	Preserved in pure ethanol
100	DysB-1	*D. brevipes*	sample reserve	Preserved in pure ethanol
101	DysB	*D. brevipes*	donated by other labs	Preserved in pure ethanol
102	DysB-3	*D. brevipes*	donated by other labs	Preserved in pure ethanol
103	PlanL-1	*Planococcus lilacinus*	sample reserve	Preserved in pure ethanol
104	PlanoL	*P. lilacinus*	sample reserve	Preserved in pure ethanol
105	PlanL-3	*P. lilacinus*	sample reserve	Preserved in pure ethanol
106	DysB-4	*D. brevipes*	sample reserve	Preserved in pure ethanol
107	Dp-1	*Dysmicoccus* sp.	sample reserve	Preserved in pure ethanol
108	Dp-2	*Dysmicoccus* sp.	donated by other labs	Preserved in pure ethanol
109	Dp-3	*Dysmicoccus* sp.	donated by other labs	Preserved in pure ethanol
110	INSV	*Orthotospovirus impatiensnecromaculae* (unknown leaf powder)	commercial suppliers	store in -80 °C
111	TRSV	*Nepovirus nicotianae* (unknown leaf powder)	commercial suppliers	store in -80 °C
112	PPV	*Potyvirus plumpoxi* (*Prunus* sp.)	sample reserve	store in -80 °C
113	TBRV	*Nepovirus nigranuli* (unknown leaf powder)	commercial suppliers	store in -80 °C
114	ToRSV	*Nepovirus lycopersici* (unknown leaf powder)	commercial suppliers	store in -80 °C
115	CGMMV	*Tobamovirus viridimaculae* (unknown leaf powder)	commercial suppliers	store in -80 °C
116	BPMV	*Comovirus siliquae* (*Glycine max*)	sample reserve	store in -80 °C
117	ToBRFV	*Tobamovirus fructirugosum* (*Capsicum annuum*)	sample reserve	store in -80 °C
118	ToBRFV-2	*T. fructirugosum* (*Capsicum annuum*)	sample reserve	store in -80 °C
119	BPMV-2	*C. siliquae* (*Glycine max*)	sample reserve	store in -80 °C
